# Eccrine Nevus Presenting with Umbilical Discharge: A Case Report and Review of the Literature

**DOI:** 10.1155/2017/9761843

**Published:** 2017-03-21

**Authors:** Farahnaz Bidari-Zerehpoosh, Shahram Sabeti, Farid Arman, Hania Shakeri

**Affiliations:** Shahid Beheshti University of Medical Sciences, Tehran, Iran

## Abstract

Eccrine nevus is a rare skin lesion with protean manifestations like hyperhidrosis, discolored nodules, papules, and so forth, which has been reported in various anatomic parts of the body including the forearm, leg, thigh, back, and coccyx. Our patient was a 26-year-old male, who presented with increasing colorless and odorless episodic umbilical discharge. First impression for the patient was an umbilical sinus and the patient underwent surgery. Histopathological study revealed the lesion to be an eccrine nevus of the umbilicus. This is the first case of eccrine nevus presenting with umbilical discharge. We recommend that eccrine nevus should be considered as a differential diagnosis for umbilical discharge.

## 1. Introduction 

Eccrine nevus is a rare skin lesion with protean manifestations [1]. It may present in childhood or adolescence with signs and symptoms such as localized unilateral hyperhidrosis with tendency to involve extremities [2] but has been reported in other anatomic locations as well [1]. It can also present with discolored nodules, papules, and plaques. Hyperhidrosis is the most commonly reported symptom, but it is not always present. Typical histopathology of eccrine nevus includes an increase in the number or size of eccrine coils [3]. Sometimes, ductal hyperplasia and dilation of lumina are notable as well [4]. Two distinct variants of eccrine nevus have been described in the literature. One of them, Eccrine Angiomatous Hamartoma (EAH), contains numerous capillary channels as well as small nerves and sometimes mucin, fatty tissue and pilar structures in between sweat gland coils, which may explain the pressure sensitivity seen in these lesions [2]. The second (often-debated) variant issudoriparousangioma is very similar to EAH, with their only difference being the presence of large caliber vessels and dilated eccrine glands in the former.

## 2. Case Report

**Figure 1:**
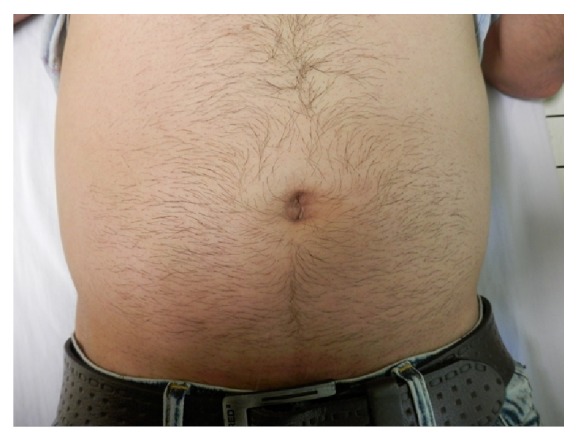
Preoperation picture.

**Figure 2:**
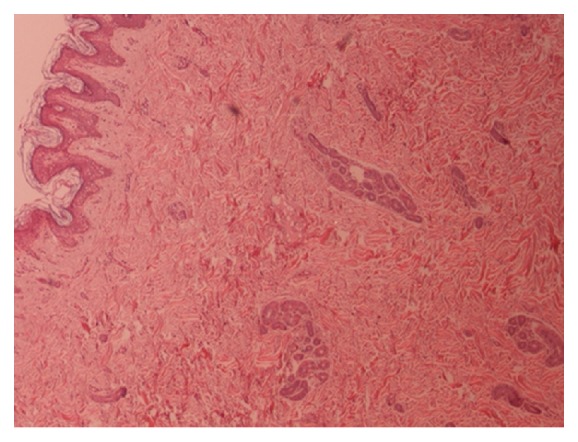
Polypoid lesion protruded to umbilicus covered by acanthotic epidermis.

**Figure 3:**
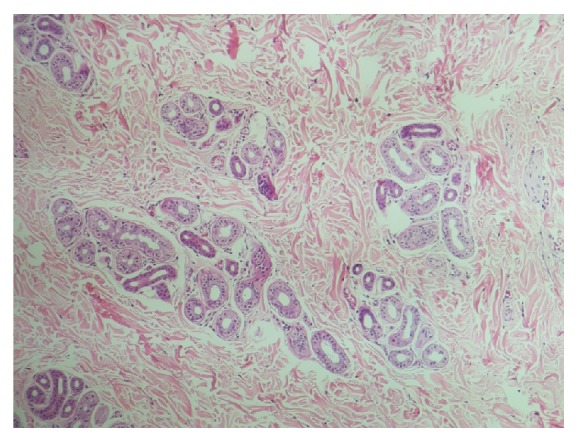
Prominent increase in eccrine coils and dermal fibrosis.

We present a 26-year-old male with the chief complaint of umbilical discharge. The problem had started approximately 7 years ago with a gradual increase in the volume and frequency of colorless, odorless, watery-serous discharge. The discharge was recently happening several times a day, with each course lasting 10 to 15 minutes. The patient noted no exacerbating factor for the discharge and no umbilical discoloration or pain. He used to dry this discharge and manipulate the umbilicus with a towel several times a day. The past medical history was insignificant. On physical exam, he had normal vital signs with no fever. In abdominal exam, no erythema, tenderness, or odor was appreciated. Umbilicus was normal looking (Figure 1). However, a deep-seated nodule was found in the umbilicus with the clinical impression of “umbilical sinus.” He underwent surgery and excisional biopsy; histopathologic examination revealed a polypoid lesion protruded to umbilicus covered by acanthotic epidermis (Figure 2) with a prominent increase in eccrine coils and dermal fibrosis (Figure 3). There was no increase in vessels and the lesion was ultimately diagnosed as “eccrine nevus.”

## 3. Discussion

Umbilical discharge can be the result of acquired pathologies, such as pilonidal sinus of umbilicus (suggested as the most common cause in one study), acute omphalitis [5], or congenital anomalies [6], but eccrine nevus and its variants have never been reported as a cause of umbilical discharge.

Eccrine nevus is a rare lesion, with 20 cases reported till 2004, reviewed by Vázquez et al. [4]. Twelve of these cases were female, 3 of them were congenital, and the oldest reported case was 79 years old. The main clinical presentation was hyperhidrosis, followed by linear papules, pigmented/depressed patches, and nodules. The most common site of involvement was forearm. One of the reported cases was an umbilical eccrine nevus in a 32-year-old female presenting with a depressed nodule [4].

Another review by Dr. Tempark and Shwayder [7] included 10 cases of mucinous eccrine nevus (MEN), 6 females and 4 males, with an age spectrum of 4 months to 57 years, and wide distribution of lesions on the leg, thigh, wrist, lower back, and other sites. Four patients presented with hyperhidrosis and 5 of them complained of pain.

Other interesting cases include a 3-month-old male with a congenital midline scalp lesion [8], an 11-year-old female with a wide area of hyperhidrosis on the right arm [9], and a 14-month-old male with a slow growing asymptomatic hypopigmented patch on his back since 3 months of age [10]. A summary of known cases is presented in Table 1.

Etiology of eccrine nevus is unknown. The proposed pathogenic factors include congenital defects in embryogenesis, trauma, and stress, but none of them has been proven [7].

Treatment of eccrine nevus can be very challenging. Different methods have been used including surgery [4], antidepressants, anticholinergics, botulinum toxin, iontophoresis, and Glycopyrrolate pad [9].

## 4. Conclusion

To the best of our knowledge, this is the second case of umbilical eccrine nevus and the first case of this kind with the chief compliant of umbilical discharge.

Thus, we recommend that eccrine nevus should be considered as a differential diagnosis of patients presenting with odorless, colorless, watery, nonbloody umbilical discharge, after excluding fistula, sinus, and infectious process. We strongly recommend a biopsy in all cases similar to our case.

## Figures and Tables

**Table 1:** Summary of reported cases of eccrine nevus in literature.

Age	Congenital, 79 years old

Gender	18 females (60%)
	12 males (40%)

Location	Upper extremity: 13 (43%)
	Lower extremity: 9 (30%)
	Trunk: 4 (13%)
	Head: 4 (13%)

Presenting signs and symptoms^*∗*^	Hyperhidrosis: 12 (40%)
	Asymptomatic: 12 (40%)
	Tenderness: 5 (16%)
	Itching: 1 (3%)

^*∗*^Asymptomatic cases were diagnosed only based on appearance and included papules, patches, and plaques.
